# A Case of Intramural Adenosarcoma With Sarcomatous Overgrowth Associated With Adenomyosis and Endometriosis

**DOI:** 10.7759/cureus.88422

**Published:** 2025-07-21

**Authors:** Kyohei Kitamura, Sachiko Minamiguchi, Hiroaki Ito, Yosuke Yamada, Yuki Himoto, Koji Yamanoi, Ken Yamaguchi, Hironori Haga

**Affiliations:** 1 Department of Diagnostic Pathology, Kyoto Katsura Hospital, Kyoto, JPN; 2 Department of Diagnostic Pathology, Kyoto University Hospital, Kyoto, JPN; 3 Department of Diagnostic Pathology, Fujita Health University Hospital, Toyoake, JPN; 4 Department of Molecular Pathology, Graduate School of Medicine, The University of Tokyo, Tokyo, JPN; 5 Department of Diagnostic Imaging and Nuclear Medicine, Kyoto University Graduate School of Medicine, Kyoto, JPN; 6 Department of Obstetrics and Gynecology, Kyoto University Graduate School of Medicine, Kyoto, JPN; 7 Department of Obstetrics and Gynecology, Graduate School of Biomedical Sciences, Hiroshima University, Hiroshima, JPN

**Keywords:** adenomyosis, adenosarcoma, endometriosis, sarcomatous overgrowth, uterus

## Abstract

Adenosarcoma is a mixed epithelial and mesenchymal neoplasm composed of a malignant mesenchymal component and a benign Müllerian glandular component. Although the endometrium is the most common primary site, adenosarcoma can also occur in the cervix, ovaries, fallopian tubes, vagina, or other sites outside the genital tract. This report presents the case of a 59-year-old woman with intramural adenosarcoma, associated with adenomyosis and endometriosis. Initially, a 4.0-cm uterine mass was identified via magnetic resonance imaging (MRI), and a watchful waiting approach was adopted. However, the mass grew to 8.5 cm over three months. A pathological examination revealed a polypoid mass with malignant spindle cells and benign glandular epithelium, confirming adenosarcoma with sarcomatous overgrowth. The endometrium showed no abnormalities, and the tumor appeared contiguous with adenomyosis and endometriosis in the myometrium and serosa of the uterine corpus, respectively. This case highlights the importance of considering adenosarcoma in the differential diagnosis of malignant transformation from adenomyosis or endometriosis.

## Introduction

Adenosarcoma is a mixed epithelial and mesenchymal neoplasm composed of a malignant mesenchymal component and a benign Müllerian glandular component. The first case of adenosarcoma was described in 1974 by Clement and Scully [1]. Although the endometrium is the most common primary site, adenosarcoma can also occur in the cervix, ovaries, fallopian tubes, vagina, or other sites outside the genital tract. Some of these have been reported to be associated with adenomyosis or endometriosis. Those cases are so rare that it is sometimes difficult to properly diagnose them preoperatively. We herein report a case of intramural adenosarcoma associated with adenomyosis and endometriosis.

## Case presentation

**Figure 1:**
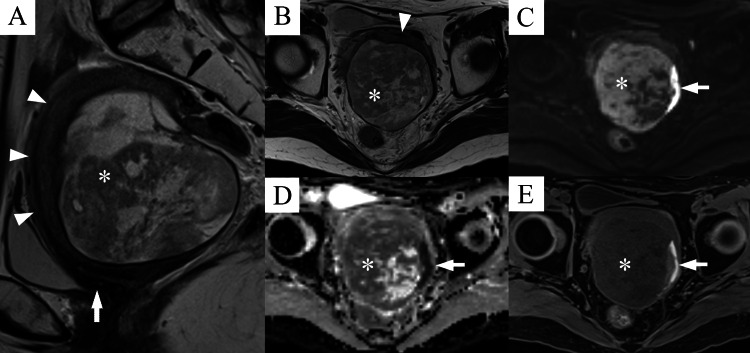
MRI findings of the tumor (A), (B) T2-weighted image of the pelvis. (A) Axial and (B) sagittal views. Arrowheads show the endometrium, and the arrow shows the uterine cervix. The tumor was located from the myometrium to the subserosa and extraserosa (asterisk). (C-E) Axial diffusion-weighted image (C), apparent diffusion coefficient (ADC) map (D), and fat-saturation T1-weighted image (E). The tumor is indicated by the asterisk. Hemorrhage was identified (arrow). (C) and (D) show heterogeneous diffusion restrictions.

A 59-year-old woman was referred to our hospital because of lower abdominal pain and a uterine mass. Adenomyosis has been previously reported. Magnetic resonance imaging (MRI) showed a 4.0-cm mass in the uterine corpus, especially in the muscle layer. Since malignancy could not be ruled out, surgical intervention was proposed. However, the patient preferred a watchful waiting approach. Approximately one month later, the patient experienced worsening abdominal pain, and three months later, follow-up MRI showed that the mass had grown from 4.0 cm to 8.5 cm. T2-weighted images showed that the tumor was located in intramuscular to sub-serosal and extra-serosal areas, away from the endometrium (Figures 1A, 1B). The tumor showed heterogeneous diffusion restriction on diffusion-weighted images and apparent diffusion coefficient maps. MRI also suggested intratumoral hemorrhage (Figures 1C-1E). Clinically, uterine sarcoma or carcinoma associated with adenomyosis was suspected. Abdominal total hysterectomy, bilateral salpingo-oophorectomy, and partial resection of the sigmoid colon were performed.

**Figure 2:**
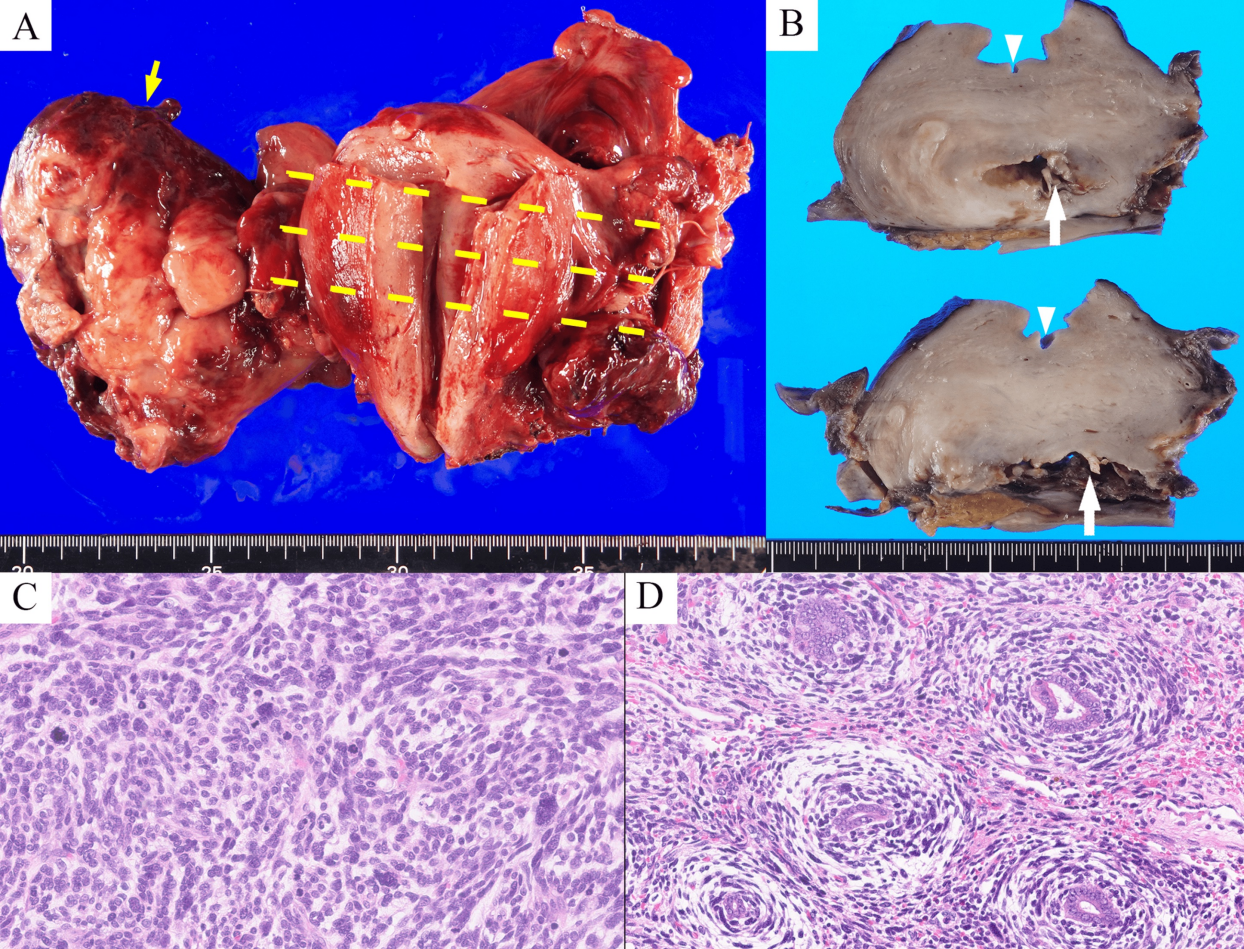
Macroscopic and microscopic findings of the tumor (A) Gross examination shows an 11.0 x 8.0 x 3.5 cm polypoid tumor polypoid mass protruding from the myometrium toward the outer surface of the posterior and right lateral uterine wall (yellow arrow). (B) Cut surface (yellow dotted line in A): No abnormality is identified in the endometrium (arrowhead). A 2.7-cm cystic cavity is observed in the myometrium of the uterus (white arrow). (C) Microscopically, the polypoid mass is entirely composed of malignant spindle cells with nuclear pleomorphism. (D) In the high-power field, malignant spindle cells show periglandular cuffing.

A gross examination of the uterus showed an 11.0×8.0×3.5-cm polypoid mass protruding from the myometrium toward the outer surface of the posterior and right lateral uterine wall (Figure 2A). The cut surface was solid and yellowish-white, with hemorrhage at the margins. In the uterine corpus, although there was no abnormality in the endometrium, a 2.7-cm intramural cystic cavity was observed, which extended outside the uterus and was continuous with the tumor (Figure 2B).

Microscopically, the polypoid mass consisted entirely of malignant spindle cells with nuclear pleomorphism (Figure 2C). The mitotic count was 15/mm^2^ and necrosis was present. In the intramural cystic cavity, the tumor showed a phyllode-like architecture with a benign glandular epithelium and a malignant mesenchymal component. Malignant spindle cells showed characteristic periglandular cuffing under high-power fields (Figure 2D). Immunohistochemically, the tumor was positive for ER and partially positive for CD10, and p53 was overexpressed. The tumor was negative for cyclin D1, desmin, alpha SMA, myogenin, and BCOR. Based on these findings, the patient was diagnosed with adenosarcoma with sarcomatous overgrowth. Disease-specific findings were not observed in the endometrium. There was no involvement of the colon, bilateral adnexa, or cervix.

**Figure 3:**
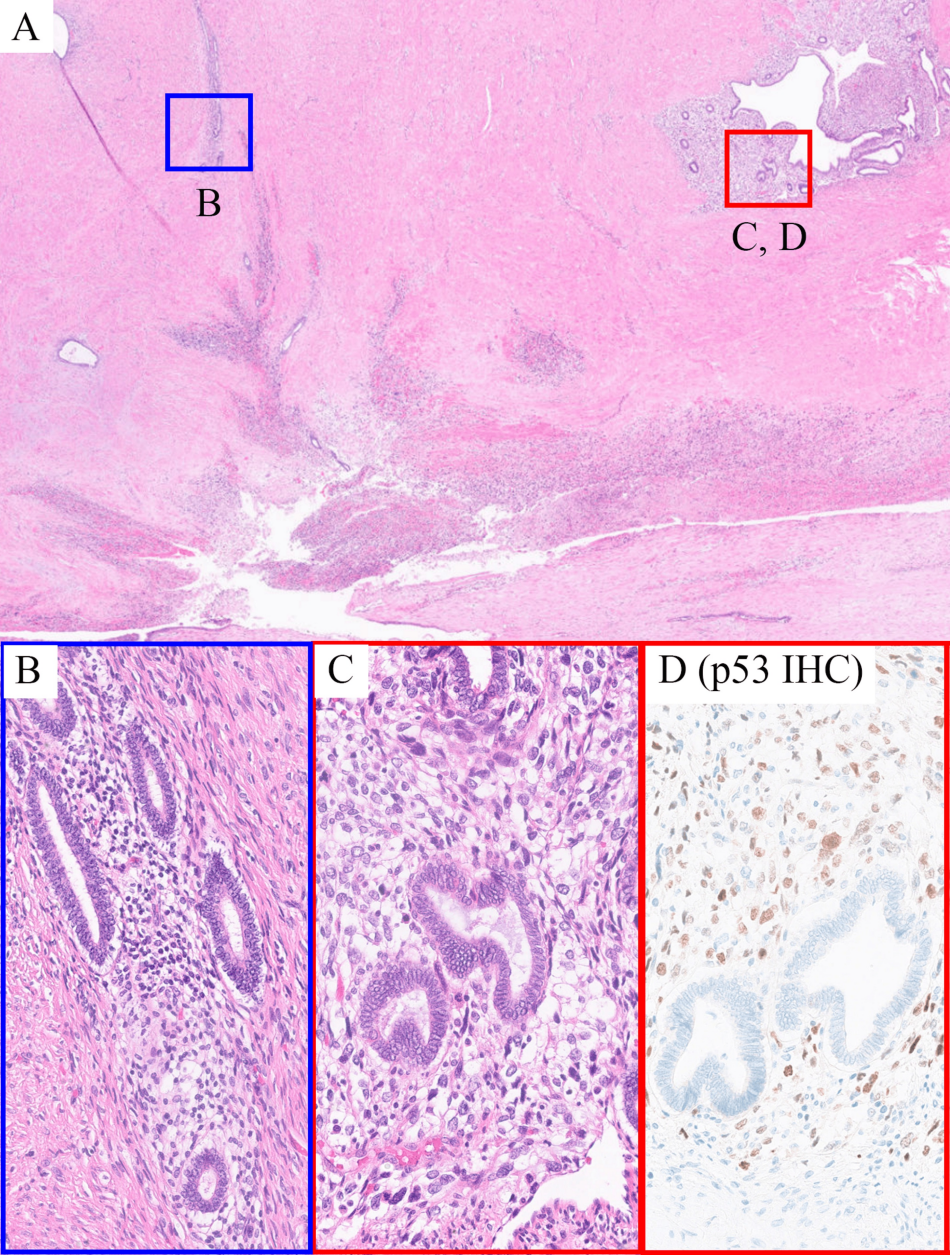
Adenosarcoma, adenomyosis, and endometriosis (A) In a low-power field, adenomyosis/endometriosis (blue squares) can be observed around the tumor (red squares). (B) Adenomyosis/endometriosis. (C) Adenosarcoma; malignant spindle cells proliferate with benign glandular components. (D) Immunohistochemically, p53 is overexpressed in the tumor cells of C.

Non-neoplastic glandular epithelium was observed in the myometrium and serosa of the uterine corpus. This was ectopically located endometrial tissue, which was consistent with adenomyosis and endometriosis. There was infiltrative growth of tumor cells around them (Figures 3A-3C). The cells were differentiated using p53 immunohistochemistry. Immunohistochemistry for p53 showed overexpression in adenosarcoma and wild-type expression in adenomyosis and endometriosis (Figure 3D). The tumor, adenomyosis, and endometriosis appeared to be contiguous. This finding suggests that the tumor was associated with adenomyosis and endometriosis.

Because this case exhibited sarcomatous overgrowth, the patient was treated postoperatively with doxorubicin alone. However, approximately five months after surgery, radiological imaging revealed multiple nodules in the pelvis, which were suspected to represent recurrence. The patient subsequently developed small bowel obstruction, and surgical resection of the pelvic mass was performed. Histopathological examination confirmed a high-grade sarcoma, consistent with recurrence. Then the patient was treated with ifosfamide and cisplatin. The treatment showed a marked effect, achieving a near-complete response on imaging. Four months later, the pelvic lymph nodes and pelvic masses enlarged again. Given the prospect of prolonged treatment, eribulin was selected as the next line of therapy. As it was ineffective, however, the regimen was switched back to ifosfamide and cisplatin, which is still being administered. Moreover, palliative radiation therapy was carried out and achieved a therapeutic effect.

## Discussion

Adenosarcoma is a biphasic neoplasm composed of benign epithelial and malignant stromal components. This tumor accounts for 5%-10% of all uterine sarcomas. While it is most often seen in perimenopausal and postmenopausal women (median age, 50-59 years), it can occur at all ages (range 15-90 years) [2].

Clinically, the most common symptom is abnormal vaginal bleeding. Other common symptoms include vaginal discharge, abdominal pain, nonspecific urinary symptoms, and palpable pelvic mass [2]. In our case, because the tumor arose not in the endometrium but in the myometrium, the patient did not show vaginal bleeding.

From a pathological point of view, the tumor shows phyllodiform cleft-like or dilated glands lined by benign endometrial or ciliated epithelium, surrounded by a distinct cuff of neoplastic stroma (periglandular cuffing). The sarcomatous component is most often the nondescript homologous type, but rhabdomyosarcomatous differentiation is possible [3]. The sarcomatous component may overgrow the epithelial component, referred to as sarcomatous overgrowth. Adenosarcoma with sarcomatous overgrowth is defined as an adenosarcoma in which the sarcomatous component constitutes more than 25% of the tumor. Adenosarcomas with sarcomatous overgrowth are found in 8-54% of cases [4].

Adenosarcomas associated with adenomyosis or endometriosis are very rare and have only been described in a small number of case reports [5-7]. In these reports, the age of onset was mainly in the 30s to 50s, with a few cases in which adenomyosis or endometriosis was noted preoperatively on imaging. The incidence of adenosarcoma arising from endometriosis is reported to be 0.3% in patients with endometriosis [8].

MRI findings of adenosarcoma in the endometrial cavity typically present as solitary, exophytic, polypoid masses. The mass consists of heterogeneous solid components with tiny cysts corresponding to glandular cavities that show high signal intensity on T2-weighted images. The solid component shows a heterogeneous high signal intensity relative to the myometrium on T2-weighted images. Hemorrhagic necrosis with high signal intensity on T1-weighted images is common, particularly when sarcomatous overgrowth is present [9]. Reports of MRI findings of intramural adenosarcomas are rare. Some studies have shown that intramural adenosarcoma exhibits similar features to adenosarcoma in the endometrium [10].

In terms of the pathological differential diagnosis, it is important to distinguish adenosarcoma from other uterine sarcomas such as endometrial stromal sarcoma and leiomyosarcoma. Considering the histopathological features, periglandular cuffing is key to the diagnosis. Immunohistochemistry is also useful for differentiating adenosarcoma from other types of sarcomas. Adenosarcomas are often positive for CD10, ER, and PR, and adenosarcomas with sarcomatous overgrowth frequently exhibit abnormal p53 expression. In the present case, the histopathological and immunohistochemical features were typical of adenosarcoma, although the tumor had grown intramurally. To summarize the differential diagnoses in this case, uterine sarcoma and carcinoma associated with adenomyosis were suspected clinically, and other types of sarcomas were the main pathological differential diagnoses.

There are some reports on the prognosis of adenosarcomas arising in the endometrium. The prognosis is generally favorable in comparison to other gynecological sarcomas. Sarcomatous overgrowth is a predictor of worse progression-free survival (PFS) and overall survival (OS). It is reported that the median PFS and OS are 29.4 and 55.4 months, respectively, in patients with sarcomatous overgrowth, compared to 105.9 and 112.4 months in patients without sarcomatous overgrowth [11]. Moreover, lymphovascular invasion, necrosis, and the presence of heterologous elements, including rhabdomyoblastic differentiation, are prognostic markers [4]. Few reports have described the prognosis of adenosarcoma arising from adenomyosis or endometriosis. Some studies have suggested that adenosarcoma arising in endometriosis has a favorable prognosis [12]. In our case, the tumor recurred five months after surgery, suggesting a poor prognosis. This is probably due to the presence of sarcomatous overgrowth. However, ifosfamide and cisplatin therapy was highly effective. Further investigation is required regarding prognosis and treatment strategies.

## Conclusions

This report describes a case of uterine intramural adenosarcoma associated with adenomyosis and endometriosis. Adenosarcomas associated with adenomyosis or endometriosis are very rare. However, as demonstrated in this case, both carcinoma and adenosarcoma should be considered in the differential diagnosis of malignant transformation from adenomyosis or endometriosis. While the prognosis of adenosarcoma associated with adenomyosis or endometriosis is not well defined, the presence of sarcomatous overgrowth, as in our case, can indicate a poorer prognosis.
